# Supplementary material for: Gender-based violence care in Mauritania: Experience and caseload of six specialized hospital units (2018–2023)
Source: PLOS Glob Public Health. 2024 Aug 1;4(8):e0003410. doi: 10.1371/journal.pgph.0003410 (PMC11293728; doi:10.1371/journal.pgph.0003410)
Supplement: S2 File — (PDF) [file pgph.0003410.s002.pdf]

Dataset description

Parameter	Possible values	Description
**USPEC Consult**	CHME	*Centre Hospitalier Mère-Enfant, Nouakchott*
	CHA	*Centre Hospitalier de l'Amitié, Nouakchott*
	CHSBY	*Centre Hospitalier de Sélibaby*
	CHKDI	*Centre Hospitalier de Kaedi*
	CHKFA	*Centre Hospitalier de Kiffa*
	CHNDB	*Centre Hospitalier de Nouadhibou*
**Police requisition**	1	*Victim presents with police requisition*
	0	*Victim presents without police requisition*
	Nil	*Missing data*
**Consultation date**	[2018/01/01-2023/06/30]	*Date of initial consultation*
	Nil	*Missing data*
**GBV date**	[2000/01/01-2023/06/30]	*GBV date, when precisely known*
	AAAA/01/01	*GBV date estimate, if only year is known*
	AAAA/MM/01	*BGV date estimate, if only year and month are known*
	Nil	*Missing data*
**Consultation delay**	1	*Less than 24 hours*
	2	*Less than 48 hours*
	3	*Less than 72 hours*
	4	*More than 72 hours*
	5	*Unknown*
	Nil	*Missing data*
**Sex**	1	*Male*
	2	*Female*
	Nil	*Missing data*
**Age group**	1	*0 to 5 years old*
	2	*6 to 11 years old*
	3	*12 to 17 years old*
	4	*18 or above*
	Nil	*Missing data*
**Adress**	1	*Nouakchott (urban)*
	2	*Kiffa (urban)*
	3	*Sélibaby (urban)*
	4	*Nouadhibou (urban)*
	5	*Kaédi (urban)*
	6	*Other (urban)*
	7	*Assaba (rural)*
	8	*Guidimagha (rural)*
	9	*Dakhlet (rural)*
	10	*Gorgol (rural)*
	11	*Other (rural)*
	12	*Outside Mauritania*
	Nil	*Missing data*
**Education level**	1	*None*
	2	*Quranic school*
	3	*Primary*
	4	*Secondary*
	5	*Post-graduate*
	6	*Other*
	Nil	*Missing data*
**Marital status**	1	Single
	2	*Married*
	3	*Divorced*
	4	*Widowed*
	Nil	*Missing data*
**Type of GBV**	1	*Rape (SV)*
	2	*Other, non-penetrative sexual violence (SV)*
	3	*Collective rape (SV)*
	4	*FGM*
	5	*Intimate-Partner physical violence (Priorily "conjugal violence")*
	6	*Adolescent pregnancy / child marriage (or risk thereof)*
	7	*Reproductive coercicion*
	8	*Non-IPV physical/domestic violence*
	9	*Other*
	Nil	*Missing data*

Parameter	Possible values	Description
**Location**	1	*Victim's home*
	2	*Street, shop, other public place*
	3	*Taxi, transport*
	4	*Unkwown by the victim*
	5	*School*
	6	*Other*
	7	*Other private location, including perpetrator's home*
	0	*Not disclosed by the victim*
	Nil	*Missing data*
**Reoccurence with the same perpetrator**	1	*Yes*
	2	*No*
	0	*Not disclosed by the victim*
	Nil	*Missing data*
**Perpetrator**	1	*Intimate partner (mari, petit-copain)*
	2	*Relative: father, uncle, cousin, step-parent, etc.*
	3	*Entourage (friend, neighbor, acquaintance)*
	4	*Unkown ((the victim may recognize her attacker but had never seen him before)*
	5	*Unkown (the victim does not know or does not wish to say)*
	0	*Not disclosed by the victim*
	Nil	*Missing data*
**Pregnancy**	1	*Yes - Confirmation of pregnancy (βHCG or ultrasound)*
	2	*No*
	Nil	*Missing data*
**Emergency contraception delivery**	1	*Yes*
	2	*No*
	Nil	*Missing data*
**Treatment**	1	*Local treatment*
	2	*Surgical treatment*
	3	*None*
	Nil	*Missing data*
**Hospitalization**	1	*Yes*
	2	*No*
	Nil	*Missing data*
**Hospitalization, number of days**	[0,10]	*Number of days*
	Nil	*Missing data*
