# Supplementary material for: Gender-based violence care in Mauritania: Experience and caseload of six specialized hospital units (2018–2023)
Source: PLOS Glob Public Health. 2024 Aug 1;4(8):e0003410. doi: 10.1371/journal.pgph.0003410 (PMC11293728; doi:10.1371/journal.pgph.0003410)
Supplement: Attachment — Submitted filename: Response to Reviewers.docx [file pgph.0003410.s003.docx]

Clairanne Bost

Tevragh Zeina, ilot K 118, Nouakchott, Mauritania

Coord.med.genre.mauritanie@medicosdelmundo.org

+222 49 88 15 39

Nouakchott, May 18^th^ 2024

Dear Editor,

We wish to express our gratitude for providing us with the opportunity to revise our manuscript “Gender violence care in Mauritania: experience and caseload of six specialized hospital units (2018-2023)”, and to proceed with the publication process.

We appreciate that both reviewers have provided us with in-depth understanding of our rationale and with their valuable comments. After careful consideration, we have tried our best to address them and hereby propose a revised manuscript, as well as our point-by-point response below.

**Response to Reviewer 1**

[Comment 1] *“Under the inclusion criteria in the Methods section, the authors indicated that there is no age restriction, and they have provided detailed information regarding adolescent pregnancies and child marriages. I am wondering if detailed information could also be provided on child abuse—perhaps including* *child abuse/maltreatment as a key term as well.”*

[Response Comment 1] Thank you for highlighting the importance of addressing child abuse in our study, we appreciate the suggestion and have taken it into account. In response, we have included a more in-depth approach on child abuse in the Discussion section (lines 354-357 and 445-454). Additionally, we have incorporated child abuse/maltreatment as a key term to ensure comprehensive coverage of this critical issue.

[Comment 2] *“According to the authors, approximately 78% were girls under 18 years old. I was expecting a more detailed discussion on why this is the case in this context. I believe the authors should further elaborate, such as what insights can be gained from this?”*

[Response Comment 2] Thank you for pointing out insufficient elaboration, especially as the striking proportion of adolescent girls constitutes the main finding of the study. Second Discussion paragraph (line) is dedicated to analyze this finding, but we have revised our writing to put more emphasis on the topic. In addition to insights regarding healthcare-seeking motivation increased by the risk of pregnancy, social worth attributed to adolescent girls’ virginity, plausible causes for under-representation of adult women and men and boys, your comment (as well as your first one) led us to reflect and an add a comparative analysis with other research and policy, more emphasis on children, and interconnectedness with IPV (lines 348-357). We also emphasized this finding in the conclusion (lines 511-513).

[Comment 3] *“There is need for copyediting the whole article for grammatical errors. For example, line 148 the authors wrote ‘were included all cases’ instead of ‘we included’. There are several areas with these areas, I think the authors should take some time to really copyedit the manuscript.”*

[Response Comment 3]: Revised accordingly.

[Comment 4] *“The authors should also ensure consistency in the use of acronyms such as Gender-Based Violence (GBV), Intimate Partner Violence (IPV), and World Health Organization (WHO). We use the full terms the first time we mention them and use the acronyms throughout the manuscript.”*
[Response Comment 4]: Revised accordingly.

**Response to Reviewer 2**

[Comment 1] *“Briefly mention the research question or objective within the introduction.”*

[Response Comment 1]: Revised accordingly.

[Comment 2] *“Consider adding a sentence about the sample size (number of cases) at the beginning of the "Data collection and material" section (line 165), for better context.”*

[Response Comment 2]: Since the total number of cases was obtained as a result of the study and not as a result of preliminary sample-size calculation, we left it into the result section. However, we wish to thank you for this comment, which had us realize this was insufficiently clear in our presentation. In particular, we did not show as a result the number of records included/excluded, and have corrected accordingly with an introduction of the result section (lines 213-215). We hope this will improve clarity and context.

[Comment 3] “Briefly mention the statistical methods used for data analysis in the "Data analysis" section (line 176). For example, you could state that "descriptive statistics" were used to summarize the data or that "chi-square tests" were used to compare proportions between groups, as the case may be.”

[Response Comment 3]: Thank you for pointing that out, we revised accordingly (lines 183-187)

[Comment 4] “*The discussion section thoroughly analyzes results, positions them within existing research, acknowledges limitations, and proposes future research directions. The discussion could be strengthened by including a future research direction that explores the long-term impact of the USPEC model on victims' health and well-being. This could involve following up with participants after a certain period to assess the model's effectiveness in supporting their recovery and overall well-being.”*

[Comment 5] *“The discussion transparently acknowledges limitations (selection bias, data entry errors), explains their impact (over-representing severity), and details potential consequences for the findings. Briefly mention recommendations or mitigation strategies for the identified limitations. For example, you could state that future studies might benefit from standardized data collection tools and improved training for data entry personnel.”*

[Response Comment 4 and 5]: Let me express my sincere appreciation with these two comments, which I will address together and using the first person. I agree 100% with both: as a matter of fact, I made the same observation while engaging in the data analysis and redaction process of this study, and had the chance to lead the exact improvement you suggest. As of January 2024, the USPEC teams now benefit from a digitalized database that was built on the learnings from this study, with standardized categories, a much easier interface for data entry, and, absolutely, follow-up data. Your comment provides me with an opportunity to broadcast these improvements and pave the way for the future research we will hopefully be able to conduct, and I was more than happy to add this (lines 496-502).

[Comment 6] *“The study's reliance on police referrals for data collection likely underrepresents the true prevalence of gender-based violence, particularly domestic violence and Female Genital Mutilation (FGM). Future research could address this limitation by incorporating data from additional sources, such as shelters, NGOs, and community health services.”*

[Response Comment 6]: Thank you for this important comment, to which we also absolutely agree: a major part of this study stems from our will to disseminate data and learning acquired by the NGO *Médicos del Mundo*. We have tried to showcase additional sources, insofar as permitted by PLOS Guidelines regarding gray literature, e.g. Human Rights Watch report (15). We had originally envisioned to introduce data collected by a community-based organization on FGM, though we ultimately decided against quoting these figures owing to various limits and bias in their methodology.

**Conclusion**

We sincerely appreciate the reviewers' insightful comments, suggestions and warm support. They have undoubtedly contributed to improve the quality of our manuscript, and we hope it now meets the standards of PLOS Global Health. Thank you once again for your valuable feedback and for the opportunity to revise our work. We look forward to your positive response or additional inputs.

Kind regards,

Clairanne Bost
